# Thermoluminescent composites of sintered synthetic-topaz/*in situ* corundum for dosimetry by a novel reversible process

**DOI:** 10.1016/j.heliyon.2024.e25025

**Published:** 2024-01-24

**Authors:** S.A. Sinclair, M.I. Pech-Canul, M.C. Acosta-Enríquez, R. Meléndrez Amavizca, Alejandro Sala Crist, J. Marcazzó

**Affiliations:** a Cinvestav IPN-Saltillo. Av. Industria Metalúrgica No. 1062, Parque Industrial Saltillo-Ramos Arizpe, Ramos Arizpe, Coahuila, 25900, Mexico; b Departamento de Investigación en Física, Universidad de Sonora, Blvd. Luis Encinas y Rosales S/N, Col. Centro, C.P. 83000, Hermosillo, Sonora, Mexico; c Instituto de Física Arroyo Seco (UNCPBA) and CIFICEN (UNCPBA e CICPBA e CONICET), Pinto 399, 7000, Tandil, Argentina

**Keywords:** Luminescence, Composite, Biphasic materials, Sintering, Thermoluminescence response, Dosimetry

## Abstract

Topaz (Al_2_F_1·44_(OH)_0·56_SiO_4_)/corundum (Al_2_O_3_) composites were prepared by a facile and novel reversible process from the sintering of synthetic topaz and AlF_3_ powders, with corundum formed *in situ* into the topaz matrix. The corundum formation reaction occurs in the temperature range 875–975 °C, from 40 min sintering time, obtaining the topaz- Al_2_F_1·44_(OH)_0·56_SiO_4_/corundum- Al_2_O_3_ composites. Although sintering temperature and time increment lead to higher corundum formation in the topaz matrix (78.4 wt % Al_2_O_3_ at 975 °C for 60 min), longer residence times give place to corundum percentage decrease due to topaz reconversion. The composites' microstructure is characterized by a rectangular bar with stacked pyramidal ends and polycrystals of hexagonal plates for topaz and corundum, respectively. For the topaz/corundum composites, the maximum density was 3.05 g/cm^3^ (17 % porosity) for specimens sintered at 925 °C for 20 min. The glow curves of the topaz/*in situ* corundum composite sintered at 975 °C and 0 min dwell time show thermoluminescent peaks between 180 and 250 °C, useful for dosimetric applications. The most helpful peak (at 221 °C) in the topaz/corundum composite's glow curves determined by computational deconvolution is sharp and exhibits the highest thermoluminescent response. Dose-response analysis of the composite (sintered at 975 °C for 0 min) with the best thermoluminescent response revealed two ranges of linear behavior, the first from 2 to 200 mGy, with an adjustment of 99.9 % and the second in the range 5–300 Gy (99.8 % fitting). The thermoluminescent response improvement of the topaz/corundum composites is attributed to the corundum formed *in situ* during sintering. Fading rate studies of the composite with the best sintering treatment revealed a signal decrease of 4 % after 15 days, which remained constant for up to 30 days, and 8 % after 60 days. The kinetic parameters, kinetics order (*b*), activation energy (*E*), and frequency factor (*s*) determined using the glow peak shape method showed second-order kinetics. The topaz/corundum composite with the best TL response (975 °C, 0 min) presents an effective atomic number (Z_eff_) of 11.74. The detection of lower doses (mGy) and the linear response at higher doses (Gy) of beta ^90^Sr, together with the other thermoluminescent properties, suggest that the topaz/corundum composites sintered at 975° for 0 min dwell time may find application in radiotherapy, geological dating, and environmental dosimetry.

## 1 Introduction

The use of radiation is a growing activity that has driven research into developing new or enhanced dosimetric materials. Thermoluminescent materials are used in dosimetric applications such as medical, sterilization, nuclear, water purification, sludge treatment, radionuclide, terrestrial and cosmic measurement [[1], [2], [3]]. Dosimetry is a fundamental part of quality control programs that use ionizing radiation [2], verifying the absorbed dose and comparing the measured doses with those prescribed in the standard specifications.

Thermoluminescence (TL) is one of the most employed dosimetric techniques for these studies, as it has achieved a high level of acceptance in the international scientific community since the first investigations [4]. Thermoluminescent materials applied in dosimetry should have an effective atomic number close to that of the human biological tissue (Z_eff_: 7.4), a wide linear range as a function of absorbed dose, low fading rate, reasonable sensitivity, chemical stability, and relative ease of preparation [5,6]. However, until now, no solid-state TL dosimeter fully meets all these characteristics. The only way to obtain the desired information is through a combination of various dosimetric techniques [7]. It is thus crucial to continue researching and developing new materials with enhanced properties.

Significant progress has been made by developing different materials of natural topaz [[8], [9], [10], [11]], synthetic topaz [[12], [13], [14], [15], [16], [17], [18], [19], [20], [21]], beach sand-Teflon composites [22,23], wollastonite-Teflon composites [24], citrine-Teflon [25], and natural topaz composites [1,7,[26], [27], [28]]. Recently, sintered synthetic topaz/corundum compounds – obtained by the hybrid-system chemical vapor deposition or HYSY-CVD route, developed at CINVESTAV-Saltillo [[29], [30], [31]]– with thermoluminescent behavior were reported [32]. The primary motivation for proposing composites in dosimetry is the development of materials with thermoluminescence characteristics superior to those obtained with materials of a single constituent. Improving mechanical, thermal, or luminescent behavior is possible by combining the characteristics of two or more constituents in the compound. With the composite's design philosophy, a material with exceptional properties, not attainable by constituents alone, is expected to result [33].

AlF_3_ is a material used in a wide variety of applications, including ionic conductors, protective coatings for electrodes in lithium-ion batteries, heterogeneous catalysts, and ferroelectric components, as well as applications in the chemical industry [34]. AlF_3_ has a density of 2.88 g/cm^3^; it is unstable at high temperatures and humidity since it hydrates easily with a mono- or tri-hydrate molecule, meaning that each aluminum fluoride molecule can form weak bonds with one or three water molecules. It is highly soluble in water; at room temperature, it presents a solubility of 6.7 g/L, and when hydrolyzed in the presence of water vapor, it generates undesirable gaseous products (AlOF and HF) [35].

Due to its good stability at room temperature and atmospheric pressure, and relatively low decomposition temperature (approximately 550 °C [36]), sodium hexafluorosilicate (Na_2_SiF_6_) has been used successfully in several studies to synthesize Si_3_N_4_ and Si_2_N_2_O by HYSY-CVD and HYSY-CVI (hybrid-system chemical vapor infiltration) [29,31,[37], [38], [39], [40], [41]]. In topaz synthesis by HYSY-CVD, when Na_2_SiF_6_ is heated, the silicon precursor SiF_4(g)_ is released and dragged by the inert gas flow throughout the cylindrical reaction chamber. Silicon tetrafluoride (SiF_4_) gas subsequently reacts with the Al(OH)_3_ (aluminum hydroxide) tablet as a substrate, which acts as a nucleation center to produce the stable solid topaz phase [[12], [13], [14],^32^,^33^,^37^].

To benefit from the composites' advantages, it is pertinent to conduct studies on the development of topaz/corundum composites, particularly because corundum possesses thermoluminescent behavior and enhanced mechanical properties. Accordingly, the objective of the present work is to investigate the effect of *in situ* formed corundum in topaz composites with varying sintering temperature and time on the composites’ microstructure and physical properties, establishing a correlation between the microstructure and their thermoluminescent behavior aimed at dosimetry applications.

## 2 Experimental procedure

**Fig. 1:**
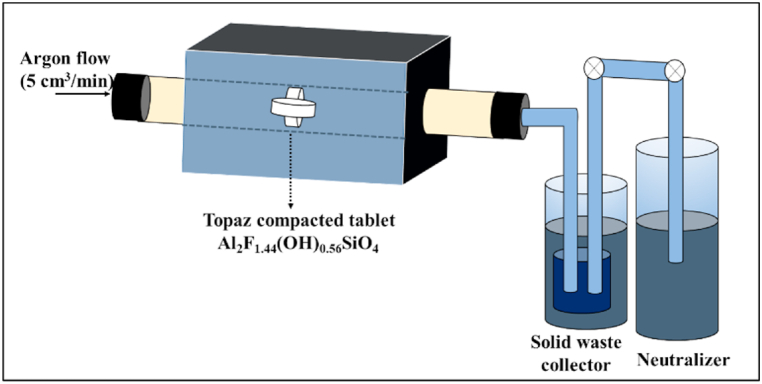
Schematic diagram of the semi-continuous cylindrical reactor used in the sintering of the topaz/corundum composite.

Topaz (Al_2_SiO_4_F_1·44_(OH)_0.56_) powders were synthesized by the hybrid system chemical vapor deposition (HYSY-CVD) method described previously [32]. HYSY-CVD is a method developed at CINVESTAV-Saltillo to produce advanced ceramics using solid-gas reaction systems [29,30,37,[39], [40], [41], [42]]. Topaz/corundum composites were prepared from the sintering of synthetic topaz powders containing AlF_3_, with the *in situ* formation of corundum in the topaz matrix. The topaz and aluminum fluoride mixture is called the biphasic material (T-F). The corundum phase proportion formed is controlled by sintering temperature and time. For this reason, adequate temperature monitoring is required with gradual increases until the desired value is reached. The topaz powders were compacted uniaxially using a Carver press model 4350L at a pressure of 14 MPa to form 1 cm diameter and 0.6 cm thick tablets and placed into the reaction chamber of a model 59,300 Thermolyne tube furnace, where the fed gas is controlled by a flow meter at the system inlet; the system's outlet is followed by a powder trap collector and by a bubbling gas phase neutralizer, as shown schematically in Fig. 1. The sintering study was performed by varying the temperature (825, 875, 925, and 975 °C) and time (0, 20, 40, 60, and 80 min) parameters, controlled by a programmable heating/dwell/cooling ramp with a rate of 15 °C/min, feeding ultra-high purity UHP argon at a flow rate of 5 cm^3^/min and maintaining the reactor pressure at 15 mbar.

Phase identification in the synthetic topaz powders and the topaz/corundum composites was conducted by X-ray diffraction (XRD) using a Phillips diffractometer model 3040 (anode excitation voltage of 40 kV and 30 mA current, monochromatic CuKα radiation (λ = 1.5418 Å) 2θ range of 10–80° at a scanning speed of 0.02°/s). Phase quantification from the diffraction patterns was performed by the XPowder® program [43]. The thermal behavior was studied using a thermogravimetric analyzer PerkinElmer model Pyris Diamond, with a sample size of 10 mg, using an argon atmosphere, flow rate of 200 mL/min, maximum temperature of 1000 °C, and heating/cooling rates of 10 and 15 °C/min. Phase morphology, distribution, and composition in the samples were analyzed by scanning electron microscopy (SEM) coupled with an energy dispersive X-ray spectrometer (EDS), using a Phillips XL30 ESEM microscope, at an accelerating voltage between 20 and 30 kV, AmpT of 51.20 and SUTW detector type Sapphire. Before the SEM examination, the specimens were coated with a layer of Au–Pd, in a piece of sputtering deposition equipment for 60 s, fixing the specimen in the sample holder with a double-sided graphite tape.

The density of the topaz/corundum composites was determined by Archimedes’ Principle using an Ohaus Explorer Pro analytical and precision balance provided with a density measurement kit. The method involves measuring the weight of the samples suspended below the surface of distilled water in a container placed on an electronic balance and measuring the same sample in the air [44]. The density of the samples was calculated with Eq. (1). The porosity of the specimens was evaluated using the ratio of the density of the samples to the theoretical density, as explained in a previous article [32].

Eq. (1) $$\rho =\frac{A}{A-B}*\rho_{o}$$

Where ρ is the sample's density (g/cm^3^), A is the weight of the sample in the air (g), B is the weight of the solid in water (g), and $\rho_{o}$ is the density of the water as a function of temperature (g/cm^3^).

The thermoluminescent characterization was conducted using a Harshaw-Bricon 3500 reader, equipped with a Hamamatsu R6094 photomultiplier tube with a sensitivity range from 300 to 650 nm, maximum sensitivity at 400 nm with ^90^Sr beta radiation source, a ratio of dose of 5 Gy/min, exposure times of 1, 5, 10, 30, 60, 120, 300, 600, 1200, 2400 and 3600 s and heat treatment from 0 to 550 °C with a heating ramp of 1 °C/s. Fading rate of the specimens was evaluated by conducting TL analyses at different periods, namely, 1, 15, 30, and 60 days after irradiation (beta ^90^Sr). The decrease in the TL signal was calculated by comparing the TL intensity of the sample measured after 1 day of irradiation with those of the different test times. During the lapse between irradiation and reading, the irradiated samples were kept at the same conditions, in a dark room at 25 °C to avoid any influence of light and to maintain signal stability. Before the first irradiation, the specimen was thermally annealed by heating from room temperature to 500C and then cooling to room temperature. For TL response measurements at low doses, ^90^Sr beta radiation source was used with doses of 2, 10, 40, and 200 mGy obtained with exposure times of 6, 30, 120, and 600 s from room temperature up to 500 °C.

## 3 Results and discussion

### 3.1 Phases, thermal analysis, and microstructure of synthetic topaz powders

**Fig. 2:**
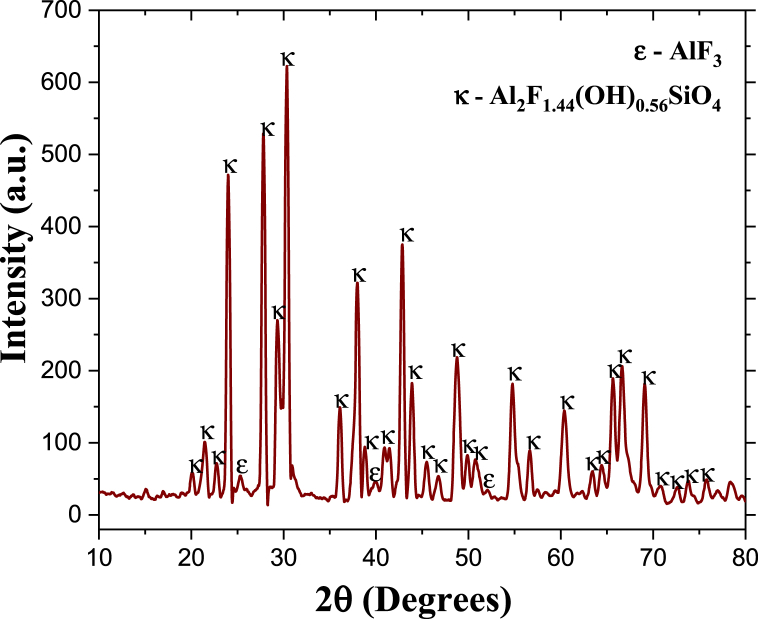
XRD pattern of synthetic topaz powders processed by HYSY-CVD (**κ-topaz, Al**_**2**_**F**_**1·44**_**(OH)**_**0·56**_**SiO**_**4**_**and ε-aluminum fluoride, AlF**_**3**_**).**

Fig. 2 shows the XRD diffraction pattern of a representative sample of topaz powders synthesized by HYSY-CVD, where the reflections correspond to topaz-Al_2_SiO_4_F_1.44_ (OH)_0.56_ (JCPDS No. 76–0480) and aluminum fluoride-AlF_3_ (JCPDS No. 80–1007). The stable synthetic topaz phase (Al_2_SiO_4_F_1·44_(OH)_0.56_) forms from a heterogeneous reaction between SiF_4(g)_ and the Al(OH)_3_ reactant compact; SiF_4(g)_ generates from the thermal decomposition of Na_2_SiF_6_ at 550 °C [29]. In parallel, as previously described, the reaction between silicon tetrafluoride and aluminum hydroxide gives rise to aluminum fluoride (AlF_3_) [32]. It is postulated that AlF_3_ formation during sintering is necessary to obtain the topaz/corundum composite.

**Fig. 3:**
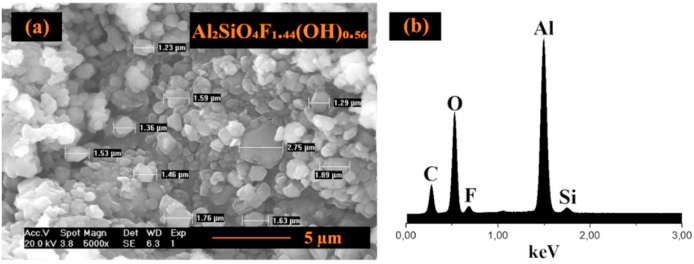
a) SEM analysis and **b)** EDS analysis spectrum of topaz powders synthesized by HYSY-CVD.

The SEM micrograph of synthetic topaz shown in Fig. 3a reveals a morphology of agglomerated and uniformly distributed hexagonal plates with an average particle size of 1.65 ± 0.44 μm. The corresponding spectrum from the EDS analysis is shown in Fig. 3b confirmed the presence of Al, O, Si, and F.

**Fig. 4:**
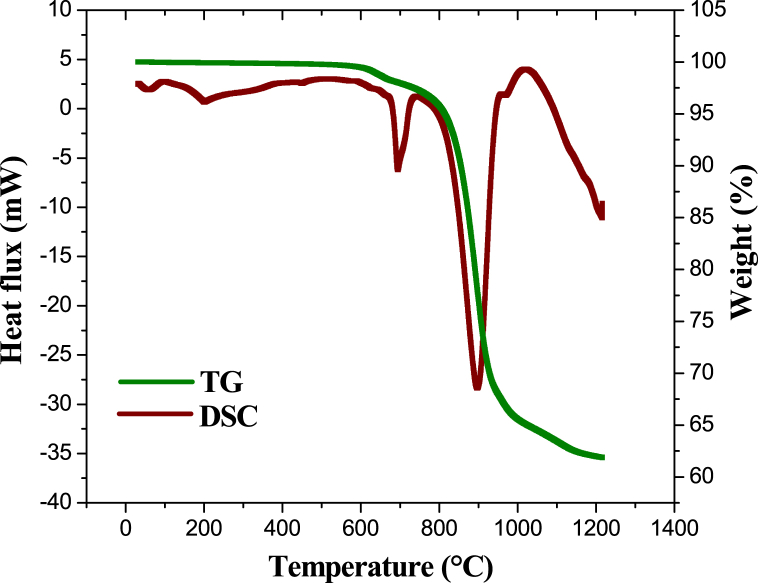
TG and DSC of a representative sample of synthetic topaz and AlF_3_ powders.

Fig. 4 shows the TG/DSC analysis results of a representative sample of topaz and aluminum fluoride powders obtained in the synthesis tests, showing two endothermic peaks related to corundum formation. As described in a previous publication [32], topaz reacts directly with AlF_3_ to form corundum (reaction (2)), and the gaseous reaction by-products, SiF_4_ and HF. These events are represented by endothermic peaks between 693 and 897 °C, with the sample's total mass loss of 38 %, due to the release of SiF_4_ and HF.

(2) $${Al}_{2}SiO_{4}F_{1.44}{\left(OH\right)}_{0.56\left(s\right)}+{1.04AlF}_{3\left(s\right)}\to 1.52{Al}_{2}O_{3\left(s\right)}+{SiF}_{4\left(g\right)}+{0.56HF}_{\;\left(g\right)}$$

### 3.2 XRD phase analysis of sintered specimens at different temperatures and dwell times

**Fig. 5:**
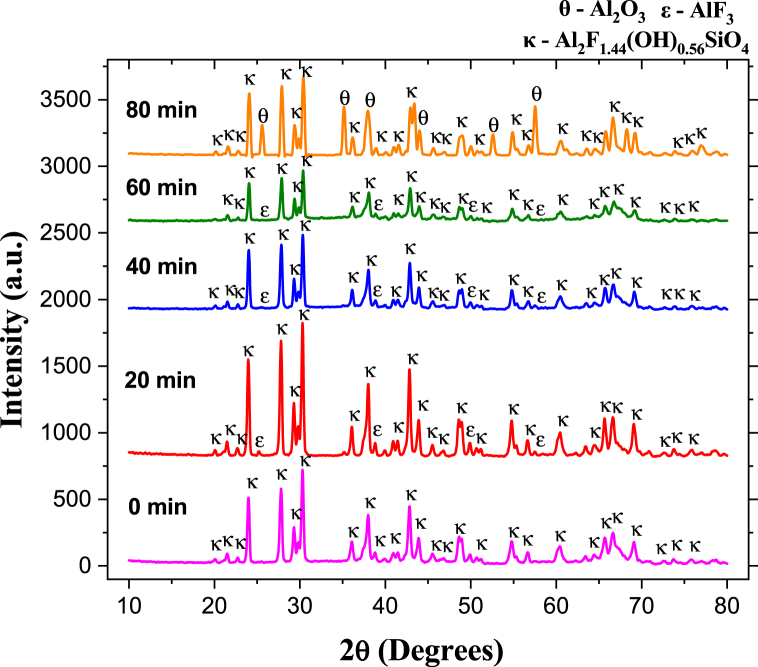
XRD pattern of biphasic material (T–F) and topaz/corundum composite sintered at 825 °C during different residence times (**κ-topaz, Al**_**2**_**F**_**1·44**_**(OH)**_**0·56**_**SiO**_**4**_, **ε-aluminum fluoride, AlF**_**3**_**and θ-corundum, Al**_**2**_**O**_**3**_**).**

**Fig. 6:**
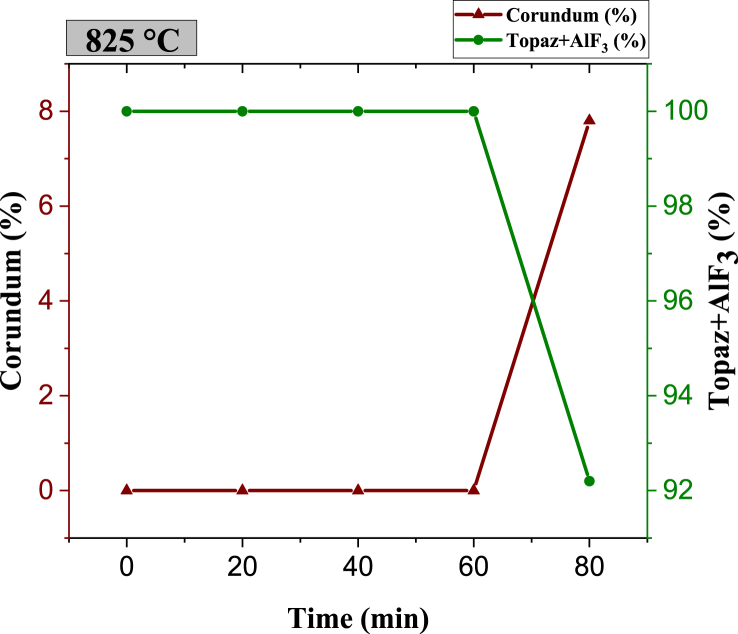
Phase quantity variation as a function of different times during sintering of specimens at 825 °C.

**Fig. 7:**
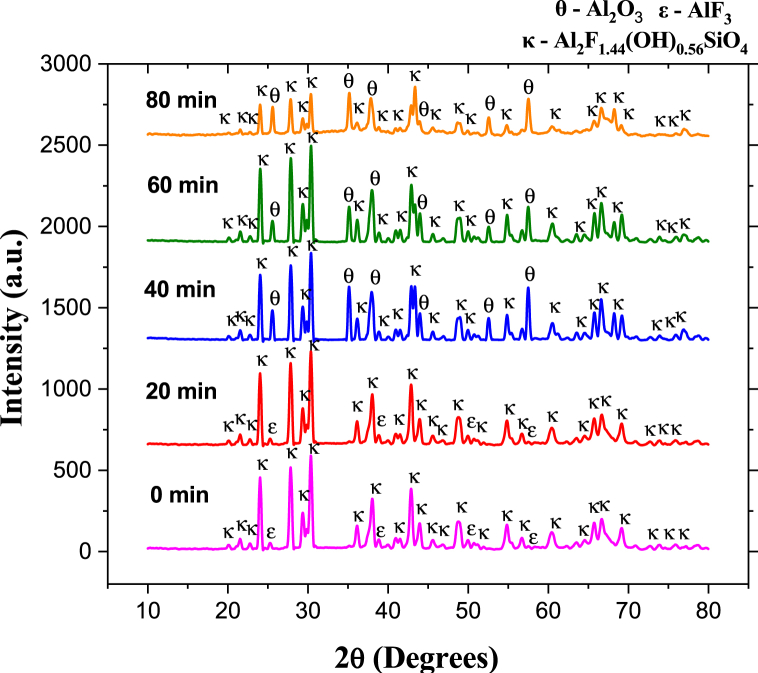
XRD pattern of biphasic material (T–F) and topaz/corundum composites sintered at 875 °C during different residence times (**κ-topaz, Al**_**2**_**F**_**1·44**_**(OH)**_**0·56**_**SiO**_**4**_, **ε-aluminum fluoride, AlF**_**3**_**, and θ-corundum, Al**_**2**_**O**_**3**_**).**

Fig. 5, Fig. 7, Fig. 9, Fig. 11 show representative X-ray diffractograms of synthetic topaz powders sintered at 825, 875, 925, and 975 °C for different dwell times (0, 20, 40, 60, and 80 min). For the specimens sintered at 825 °C (Fig. 5), the topaz-Al_2_SiO_4_F_1·44_(OH)_0.56_ (JCPDS No. 76–0480) and aluminum fluoride-AlF_3_ (JCPDS No. 80–1007) phases are identified up to 60 min. However, at 80 min, the reflections correspond to topaz-Al_2_SiO_4_F_1·44_(OH)_0.56_ and corundum-Al_2_O_3_ (JCPDS No. 43–1484) phases. Fig. 6 shows the evolution of the quantitative phase analysis as a function of time, revealing the formation of a maximum amount of 7.8 wt % corundum formed *in situ* after 80 min sintering.

**Fig. 8:**
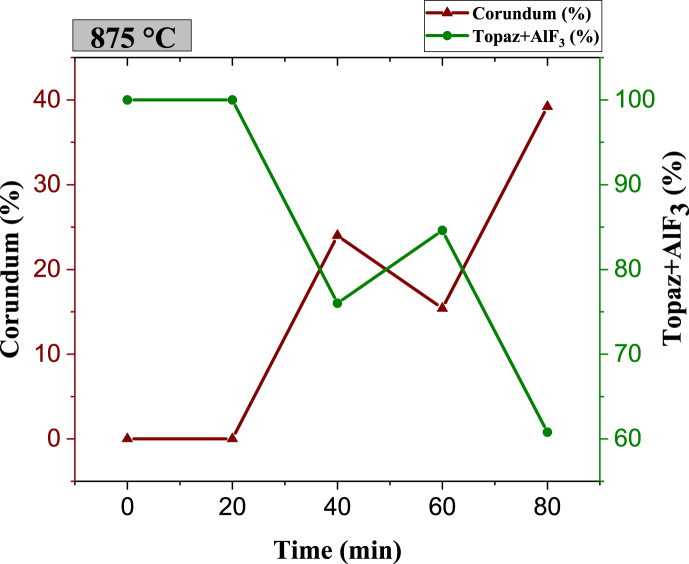
Phase quantity variation as a function of different times during sintering of specimens at 875 °C.

For specimens sintered at 875 °C, between 0 and 20 min, the XRD reflections correspond to the phases of the biphasic material (T-F), topaz-Al_2_SiO_4_F_1·44_(OH)_0.56_ (JCPDS No. 76–0480) and aluminum fluoride-AlF_3_ (JCPDS No. 80–1007). As shown in Fig. 7, after 40 min, there is evidence of corundum forming in the topaz/corundum composite. Fig. 8 shows that the highest percentage of corundum phase obtained at 80 min sintering is 39.2 wt %.

**Fig. 9:**
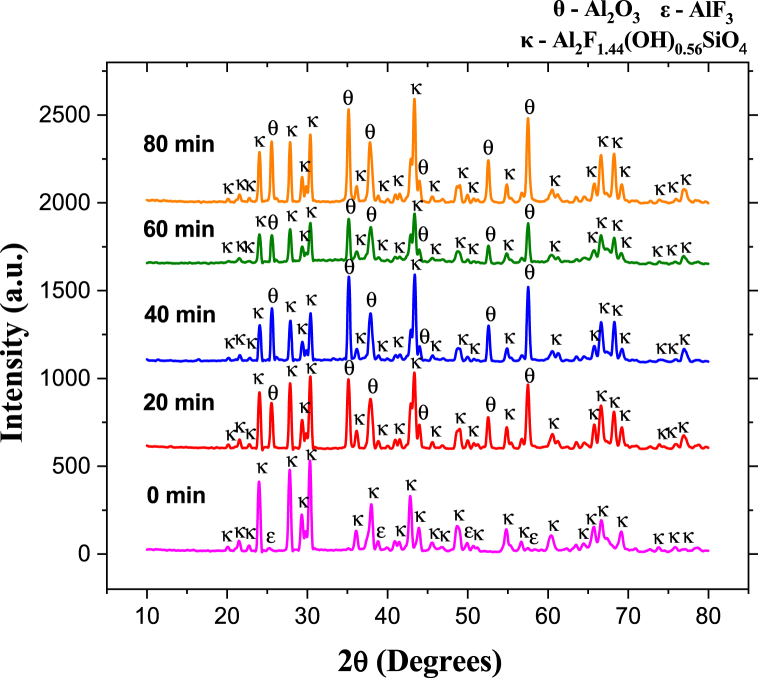
XRD pattern of biphasic material (T–F) and topaz/corundum composites sintered at 925 °C for different residence times (**κ-topaz, Al**_**2**_**F**_**1·44(**_**OH)**_**0·56**_**SiO**_**4**_, **ε-aluminum fluoride, AlF**_**3**_**, and θ-corundum, Al**_**2**_**O**_**3**_**).**

**Fig. 10:**
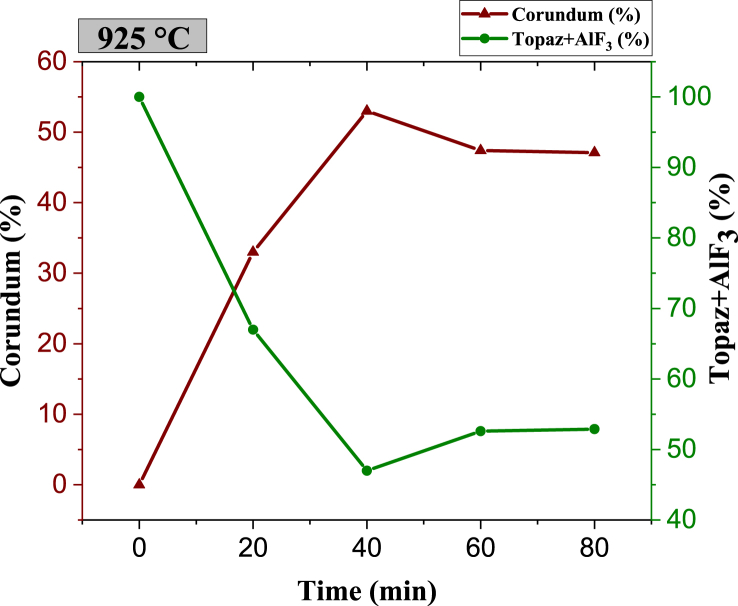
Phase quantity variation as a function of different times during sintering of specimens at 925 °C.

Higher sintering temperatures favor corundum formation at shorter times, thus producing the topaz/corundum composite. After 40 min at 925 °C, 53 wt % of *in situ* corundum phase is obtained. However, a slight decrease is observed between 40 and 60 min, remaining almost stable between 60 and 80 min, where it reaches 47 wt % (Fig. 9). Longer sintering times cause a decrease in the corundum phase percentages because of reconversion to topaz as depicted in Fig. 10. Due to the semi-continuous reactor-type configuration where corundum formation occurs, SiF_4(g)_ accumulation is produced as a reaction (1) byproduct. The retained SiF_4(g)_ reacts with the generated corundum and with water traces contained in UHP argon (<3 ppm H_2_O) for topaz reconversion, as proposed in reaction (3):

(3) $${{Al}_{2}O_{3}}_{\left(s\right)}+{{SiF}_{4}}_{\left(g\right)}+1.56{H_{2}O}_{\left(g\right)}\to {Al}_{2}SiO_{4}F_{1.44}{\left(OH\right)}_{0.56\left(s\right)}+2.56{HF}_{\left(g\right)}$$

Reactions (1) and (2) proposed suggest the recycling role of silicon tetrafluoride (SiF_4_) and topaz reconversion, depending on processing conditions. They also suggest the feasibility of engineering the composite's compositions or phase proportions in the composites.

**Fig. 11:**
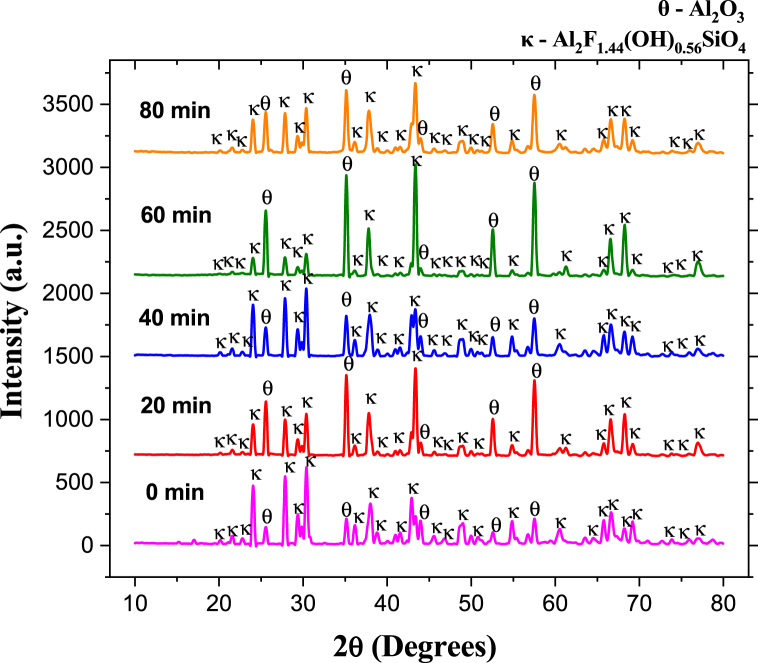
XRD pattern of topaz/corundum composites sintered at 975 °C for different residence times (**κ-topaz, Al**_**2**_**F**_**1·44**_**(OH)**_**0·56**_**SiO**_**4**_**and θ-corundum, Al**_**2**_**O**_**3**_**).**

**Fig. 12:**
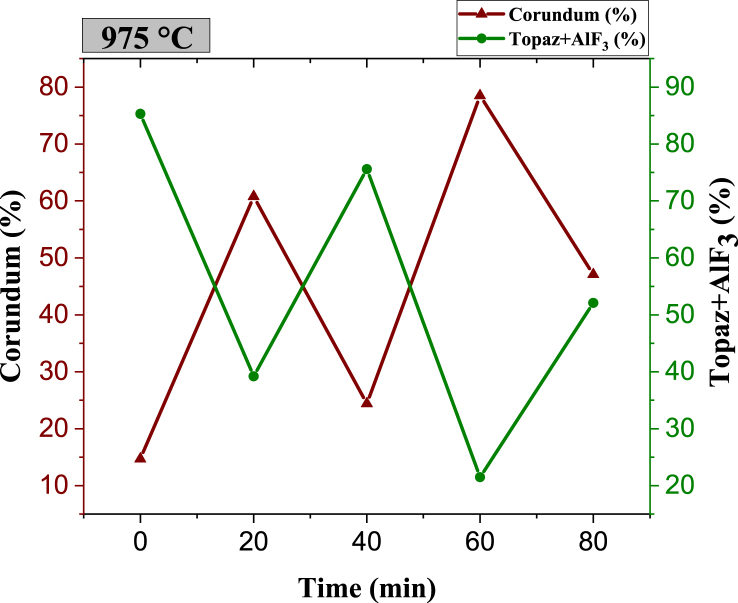
Phase quantity variation as a function of different times during sintering of specimens at 975 °C.

For the samples sintered at 975 °C and for all sintering times, the reflections in Fig. 11 correspond to topaz-Al_2_SiO_4_F_1·44_(OH)_0.56_ (JCPDS No. 76–0480) and corundum-Al_2_O_3_ (JCPDS No. 43–1484). After 20 min, corundum formation increases drastically from 14.7 wt % to 60.8 wt %, reaching the highest amount at 60 min with 78.5 wt % corundum and 21.5 wt % topaz. As time passes up to 80 min, reconversion to topaz is observed (Fig. 12), with a decrease in the corundum phase (47.1 wt %), according to reaction (2). As the temperature increases, topaz reconversion occurs at shorter times. The cyclic or wave-like behavior observed at 975 °C with corundum maxima at 20 and 60 min and with minima at 40 and 80 min suggests the potential for designing the composite's topaz/corundum proportions with specific properties for targeted applications.

### 3.3 TG/DSC of sintered topaz/corundum composites at different temperatures

**Fig. 13:**
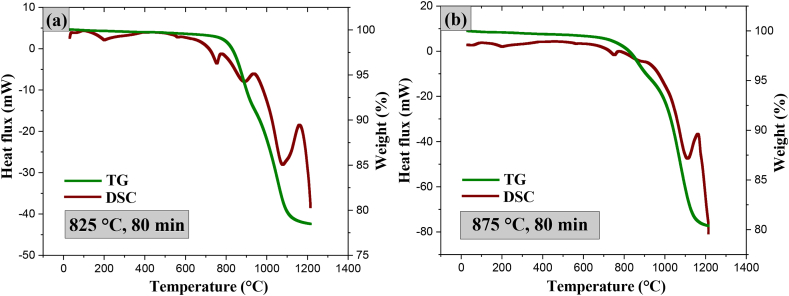
TG and DSC of topaz/corundum composites sintered at **a)** 825 and **b)** 875 °C for 80 min.

To determine phase stability, that is, to verify whether corundum formation (reaction 1) is carried out thoroughly, thermal analysis of the sintered topaz/corundum composites was carried out by heating up to 1200 °C for 80 min. For composites sintered at 825 °C (Fig. 13a) and 875 °C (Fig. 13b), at 754 °C begins a weight decrease, attributed to the release of the gaseous by-products SiF_4_ and HF, with total losses of 18.5 % and 20.84 %, respectively, corresponding to the corundum formation reaction (reaction 1)—such corundum results from the remnant reactant that did not react during composites sintering. An endothermic event is observed at 1114 °C (specimen at 825 °C) and 1079 °C (specimen at 875 °C), attributed to mullite formation, according to reaction (4) [45].

(4) $$15{Al}_{2}\left({SiO}_{4}\right){\left[{F_{0.9}\left(OH\right)}_{0.1}\right]}_{2}+2H_{2}O\to 5\left(3{Al}_{2}O_{3}*{2SiO}_{2}\right)+5{SiF}_{4}+7HF$$

**Fig. 14:**
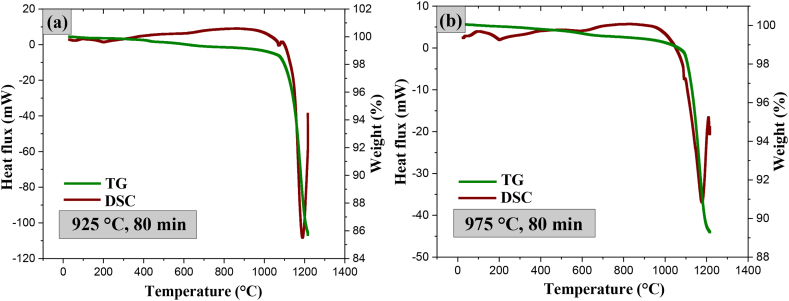
TG and DSC of topaz/corundum composites sintered at **a)** 925 and **b)** 975 °C for 80 min.

The composites sintered at higher temperatures (925 and 975 °C) show similar thermal behavior. As shown in Fig. 14a (925 °C) and Fig. 14b (975 °C), no thermal events occurred since the corundum (reaction 1) formation with SiF_4_ and HF as gaseous by-products was wholly carried out in sintering. No notable changes in the weight losses of the composites (approximately 1 %) were observed. The formation of mullite is associated with thermal events occurring at 1193 and 1181 °C, respectively, according to reaction (3). The total mass losses were 14.1 and 10.66 %, respectively. It can be concluded that the corundum formation reaction in composites sintered at 925 °C and 975 °C is complete since the composites did not show additional thermal events related to further corundum formation.

### 3.4 Density and porosity of sintered specimens at different temperatures

**Fig. 15:**
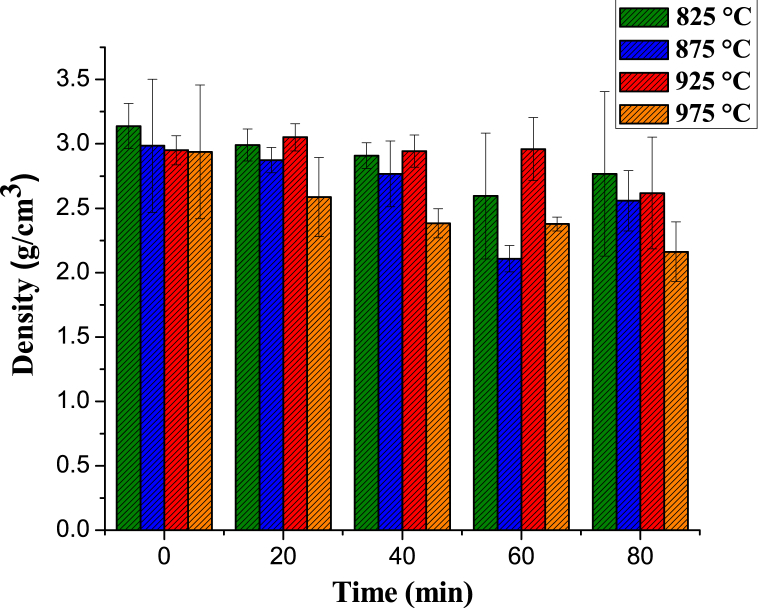
Average density graph of sintered specimens at 825, 875, 925, and 975 °C and times of 0, 20, 40, 60, and 80 min with its standard deviation.

Fig. 15 shows the results of the average density of the samples determined by Archimedes' principle for different temperatures and sintering times. The biphasic material (T-F) sintered at 825 °C for 0 min registered a maximum density of 3.13 g/cm^3^. At that temperature and sintering time, the corundum formation reaction with the release of SiF_4_ and HF gaseous by-products did not occur. The biphasic material (T-F) presented the minimum porosity (11 %), as shown in Table 1.

**Table 1:** Residual porosity of specimens sintered at 825, 875, 925, and 975 °C for 0, 20, 40, 60, and 80 min.

Time (min)	Residual porosity (%)			
	825 °C	875 °C	925 °C	975 °C
0	11	17	20	21
20	15	20	17	31
40	18	23	20	36
60	27	42	20	36
80	22	29	29	42

The highest densities obtained for 825 and 875 °C ranged from 3.13 to 2.7 g/cm^3^ between 0 and 40 min. After 60 min, a decrease in density occurs, 2.7 g/cm^3^ (for 825 °C) and 2.10 g/cm^3^ (for 875 °C). At 925 °C for 20 min, the density is 3.05 g/cm^3^, and the porosity is 17 %. At this temperature, the density remains approximately constant with increasing sintering time. At 975 °C for 80 min, the lowest density value was recorded, 2.16 g/cm^3^ and a residual porosity of 42 % was reached.

### 3.5 Microstructural evolution of sintered specimens at various temperatures and dwell times (SEM and EDS)

**Fig. 16:**
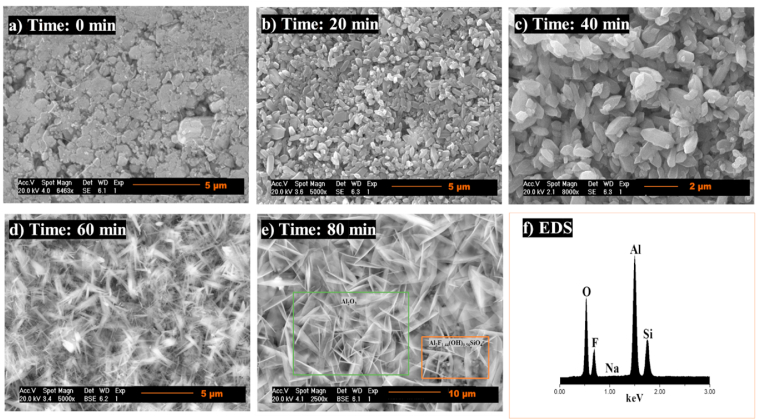
SEM analysis of topaz/corundum composites sintered at 825 °C for dwell times of **a)** 0 min, **b)** 20 min, **c)** 40 min, **d)** 60 min, **e)** 80 min and **f)** EDS analysis.

The microstructural evolution of topaz/corundum composites was examined at all sintering temperatures as a function of time (0, 20, 40, 60, and 80 min). Fig. 16 shows the micrographs of the samples sintered at 825 °C, revealing a morphology of irregular agglomerates corresponding to the topaz phase with an average particle size of 1.14 ± 0.32 μm. Compared to the micrograph of the topaz synthesized condition (Fig. 3a), the particles are in close proximity. A morphology change towards rectangular bars with pyramidal ends is observed after 20 and 40 min, with average sizes of 1.22 ± 0.28 μm and 1.11 ± 0.36 μm, respectively.

As the treatment time continues to increase, the rectangular topaz bars grow towards their ends until they reach a size of 1.96 ± 0.36 μm. At 80 min treatment, fibers corresponding to corundum uniformly distributed are observed, with an average size of 6.16 ± 1.28 μm. The EDS spectra for all analyzed samples (Fig. 16f) confirm the phases identified by XRD: Al, O, Si, and F, with a low Na content as an impurity, coming from the solid precursor Na_2_SiF_6_.

**Fig. 17:**
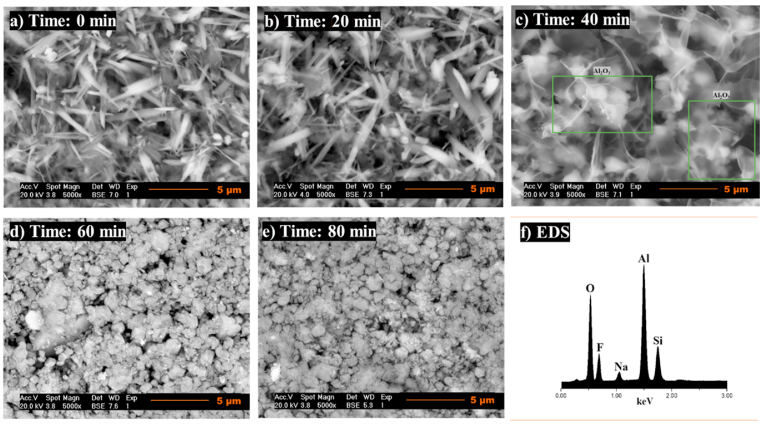
SEM analysis of topaz/corundum composites sintered at 875 °C for dwell times of **a)** 0 min, **b)** 20 min, **c)** 40 min, **d)** 60 min, **e)** 80 min and **f)** EDS analysis.

By increasing the sintering temperature to 875 °C, the *in situ* formation of corundum is observed after 40 min treatment (Fig. 17c). A morphology of rectangular bars with stacked pyramidal ends is observed, corresponding to the topaz phase, with average particle sizes between 2.74 ± 1.07 μm and 2.88 ± 1.13 μm. As time increases to 40 min, the bars grow towards their ends, where it is possible to notice the formation of corundum. Corundum's microstructure within the topaz matrix indicates that the dominant mechanism for such modification involves a corundum formation reaction with the release of the gaseous by-products SiF_4_ and HF and the bonding of the particles with the formation of the neck and shrinkage of the material. When the temperature is raised, the corundum formation reaction from topaz in the presence of aluminum fluoride is promoted, and irregular crystals are formed with a particle size of 1.48 ± 0.42 μm, uniformly distributed. An irregular corundum morphology is seen in the microstructure after 60 (Fig. 17d) and 80 min (Fig. 17e), with particles of 1.35 ± 0.40 μm and 1.09 ± 0.73 μm, respectively.

**Fig. 18:**
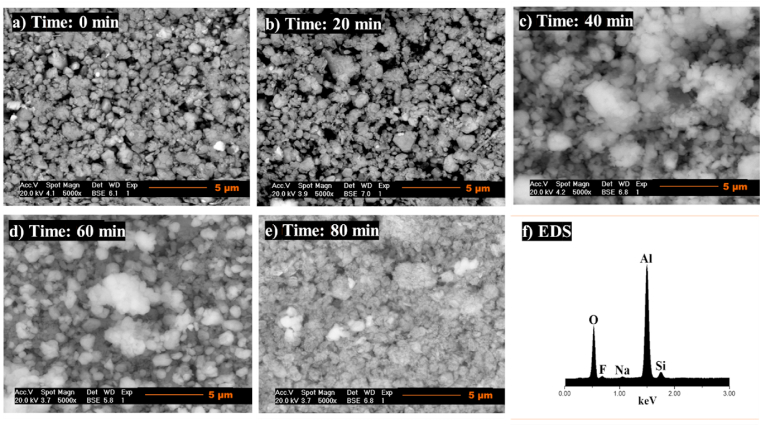
SEM analysis of topaz/corundum composites sintered at 925 °C for dwell times of **a)** 0 min, **b)** 20 min, **c)** 40 min, **d)** 60 min, **e)** 80 min and **f)** EDS analysis.

The microscopic examination of samples sintered at 925 °C shows a microstructure consisting of irregular morphology corundum uniformly distributed and in the form of agglomerates. The specimens for all sintering times exhibit average particle sizes of 1.18 ± 0.38 μm (Fig. 18b and 20 min), 1.54 ± 0.68 μm (Fig. 18c and 40 min), 1.36 ± 0.52 μm (Fig. 18d, 60 min), and 1.19 ± 0.32 μm (Fig. 18e and 80 min). The uniformly distributed corundum in the topaz phase results from the formation reaction when the temperature increases. As the sintering temperature rises, the increased corundum formation is associated with a more noticeable or growing porosity, and this is explained by fluorine, hydrogen, and silicon losses in the form of gaseous HF and SiF_4_.

**Fig. 19:**
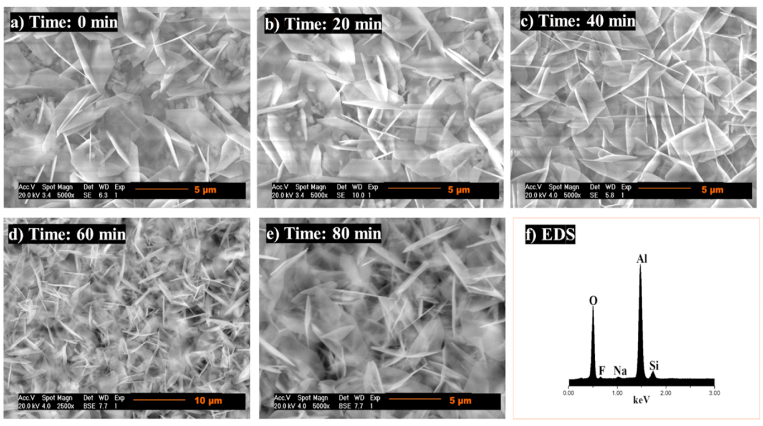
SEM analysis of topaz/corundum composites sintered at 975 °C for dwell times of **a)** 0 min, **b)** 20 min, **c)** 40 min, **d)** 60 min, **e)** 80 min and **f)** EDS analysis.

The composites’ microstructure sintered at 975 °C shows corundum polycrystals of hexagonal-plate morphology, with average particle sizes of 4.37 ± 0.97 μm (Fig, 19a, 0 min), 4.39 ± 1.52 μm (Fig. 19b, 20 min), 4.32 ± 1.17 μm (Fig. 19c and 40 min), 4.98 ± 1.12 μm (Fig. 19d and 60 min), and 4.37 ± 1.26 μm (Fig. 19e and 80 min), uniformly distributed and agglomerated. Due to its low content at this temperature and long sintering times, topaz was not detected. However, EDS analysis, shown in Fig. 19f, identifying Al, O, Si, and F confirmed the phases that were detected by XRD..

### 3.6 Thermoluminescence analysis of sintered specimens at various temperatures and dwell times

**Fig. 20:**
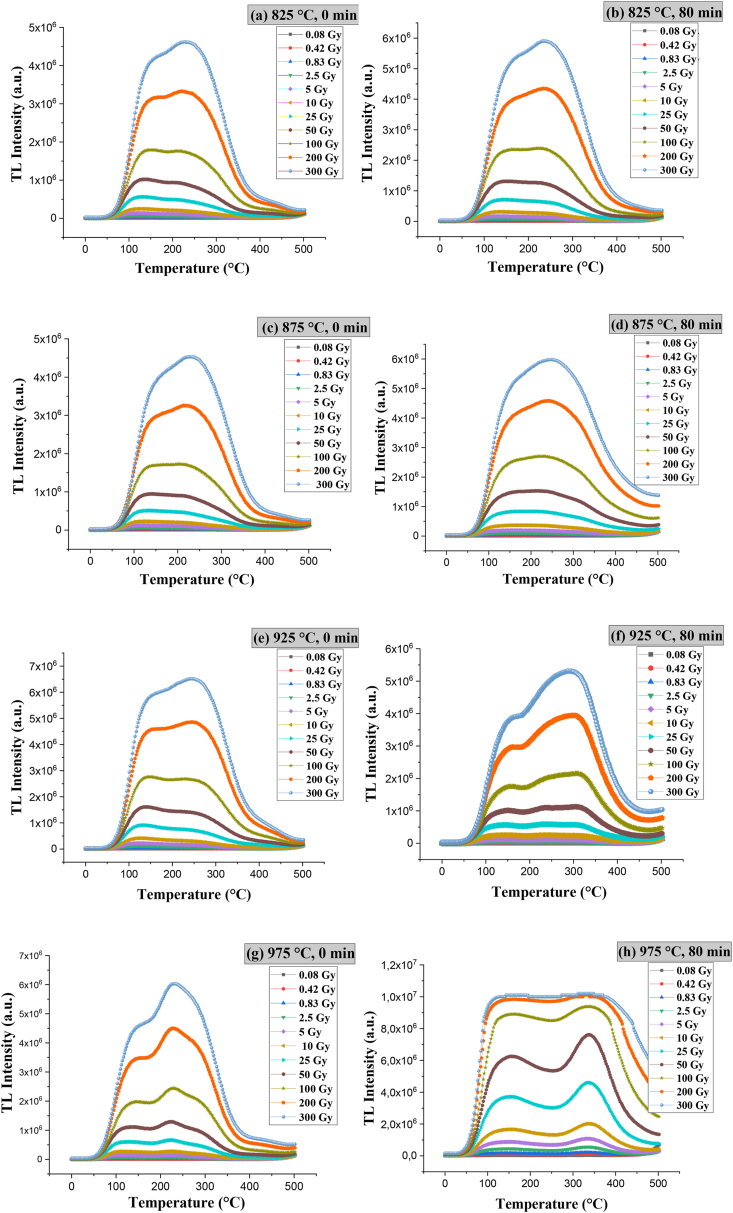
TL glow curves of specimens sintered at **a)** 825 °C for 0 min, **b)** 825 °C for 80 min, **c)** 875 °C for 0 min, **d)** 875 °C for 80 min, **e)** 925 °C for 0 min, **f)** 925 °C for 80 min, **g)** 975 °C for 0 min and **h)** 975 °C for 80 min.

**Fig. 21:**
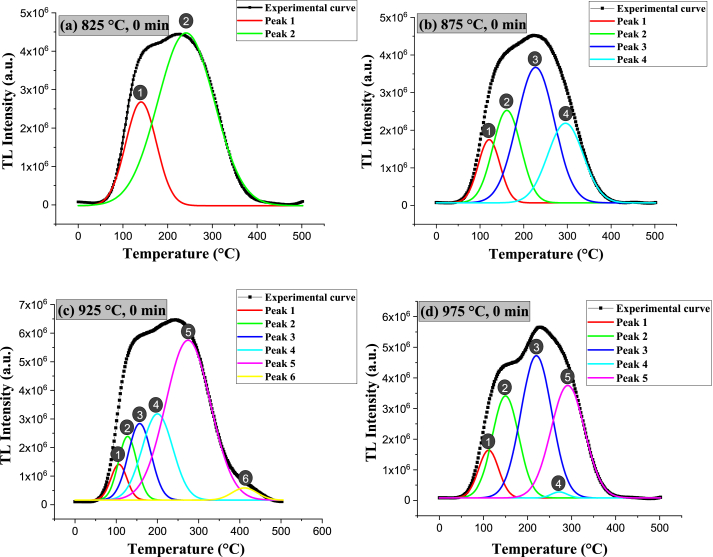
Computational deconvolution of glow curves of specimens sintered at **a)** 825 °C, **b)** 875 °C, **c)** 925 °C and **d)** 975 °C for 0 min.

Fig. 20 shows the glow curves of the samples sintered at 825, 875, 925, and 975 °C for 0 and 80 min and irradiated with beta ^90^Sr at different exposure times (1, 5, 10, 30, 60, 120, 300, 600, 1200, 2400, and 3600 s). For all samples, the intensities of the luminous peaks increase with increasing beta-ray dose as a function of temperature. This augment is because, with increasing radiation dose, the traps present and responsible for the TL peaks are occupied; the subsequent thermal stimulation releases the trapped charge carriers. To determine the number of peaks that overlap and produce the apparent TL peaks located from room temperature up to 500 °C, computational deconvolution of the original emission curves was performed, as shown in Fig. 21.

Deconvolution of the original glow curves shows that the number of peaks increases with dwell time at a given temperature. Specifically, deconvolution revealed 4 peaks at the sintering temperature of 825 °C (120, 155, 200, and 270 °C), 5 peaks at 875 °C (114, 149, 201, 297, and 426 °C), 6 peaks at 925 °C (111, 148, 210, 258, 308, and 334 °C) and 7 peaks at 975 °C (91, 114, 154, 223, 327, 397, and 441 °C) for 80 min. As the dwell time elapses, the peaks become less useful because their intensity decreases and broadens, becoming more susceptible to fading, falling outside the range for personal dosimetry application.

For all sintering temperatures (825, 875, 925, and 975 °C), the useful peaks occur at 0 min sintering time. Fig. 21 shows the glow curves’ computational deconvolution of specimens sintered at 0 min dwell time. However, the most helpful peak occurs at 975 °C (peak located at 221 °C) because it is sharp and of high intensity, indicating that more charge carriers are trapped in this type of trap. Moreover, the specimens sintered at 825, 875, and 925 °C for 0 min are topaz/AlF_3_ biphasic materials (T-F), while the one treated at 975 °C for 0 min is topaz/corundum composite (Fig. 21d). Therefore, the topaz/corundum composite with potential application for radiotherapy and geological dating dosimetry is sintered at 975 °C for 0 min, i.e., the one treated thermally just upon reaching 975 °C. Such thermoluminescent behavior is associated with two types of color centers [AlO_4_]° and [H_3_O_4_]°, emitting at 460 nm and 380 nm, respectively, presented in the topaz [46,47].

Lithium fluoride (LiF) is the most studied material with dosimetric properties, being commercialized as a dosimeter in its TLD-100 form (7 % of ^6^Li and 92.5 % of ^7^Li) with magnesium and titanium impurities at 300 and 15 ppm, respectively. The glow curves of TLD-100 consist of 5 characteristic peaks (65, 120, 140, 195, and 210 °C), where the signal of peak 5 located up to 230 °C is widely used in dosimetry [48]. The maximum intensities in the glow curves of topaz/corundum composites sintered in this work are within the most appropriate range for dosimetric applications (160 and 250 °C) [49]. Since the samples with higher corundum proportion present a higher intensity peak, the highest thermoluminescent response is attributed to the corundum formed *in situ* into the topaz matrix during sintering. It is worth mentioning that the samples maintained their physical and mechanical integrity during their handling for characterization. Considering the importance of the physical/mechanical aspects of disk-shaped composite specimens and, as an ongoing project, the results of mechanical properties will be reported elsewhere.

**Fig. 22:**
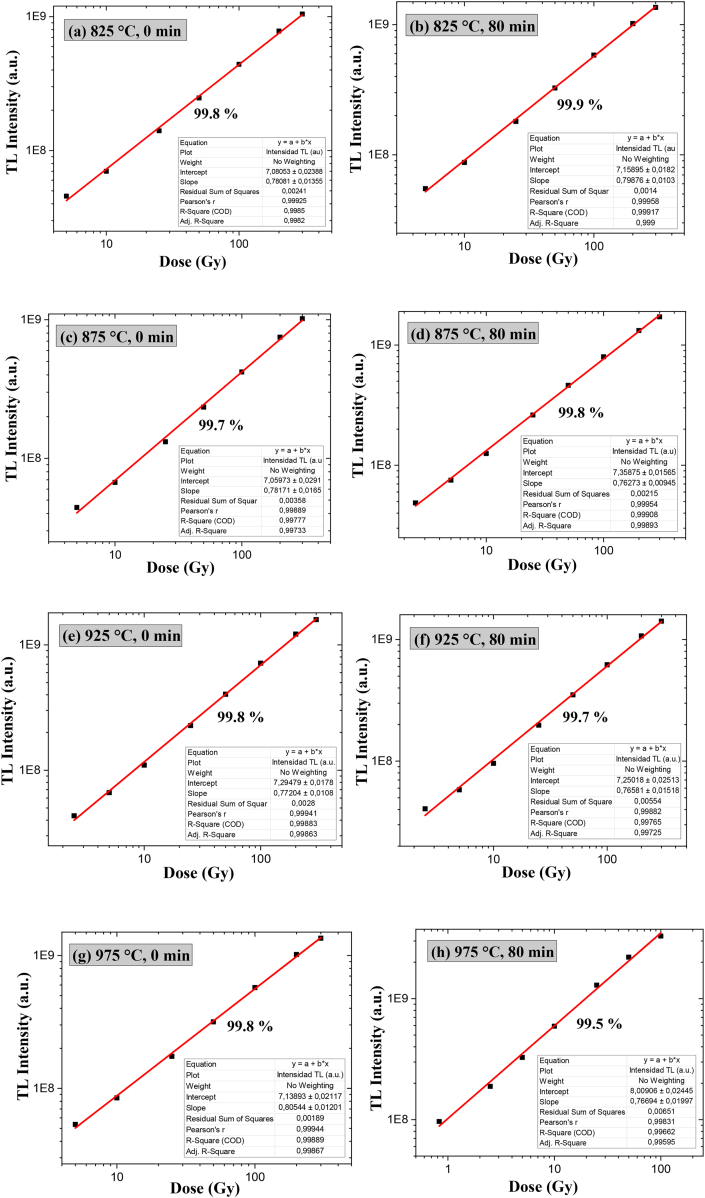
TL response as a function of the beta doses (Gy) of specimens sintered at **a)** 825 °C for 0 min, **b)** 825 °C for 80 min, **c)** 875 °C for 0 min, **d)** 875 °C for 80 min, **e)** 925 °C for 0 min, **f)** 925 °C for 80 min, **g)** 975 °C for 0 min and **h)** 975 °C for 80 min.

The graphs of TL intensity vs. beta dose (Gy) shown in Fig. 22 represent the areas under the curves of the different glow curves of the topaz aluminum biphasic material (T-F) and topaz/corundum composites exposed to ^90^Sr beta radiation. The linear response range as a function of absorbed dose varies from 5 to 300 Gy for sintering temperatures of 825, 875, and 975 °C, with settings above 99 %. When the time increased to 80 min for 975 °C, the linear response range was up to 200 Gy, with 99.5 % of adjustment.

**Fig. 23:**
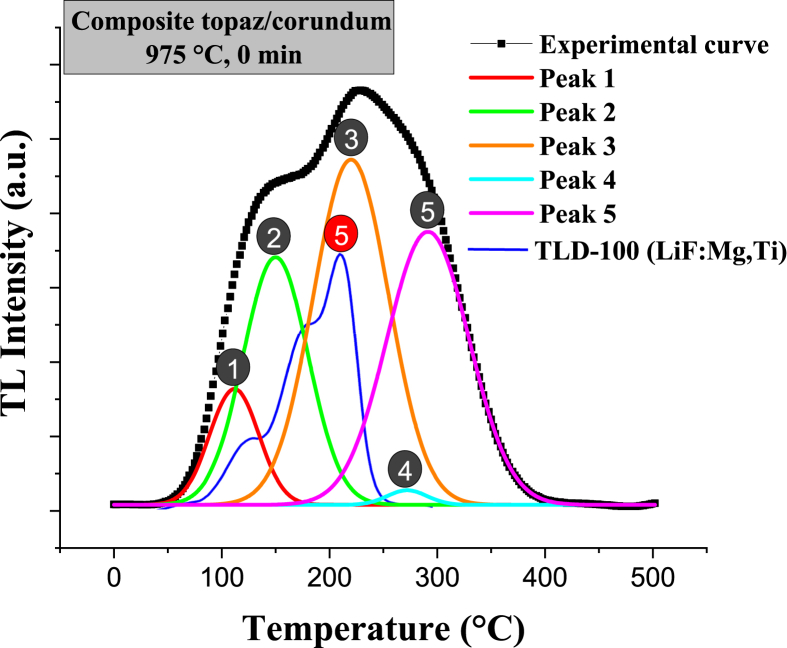
Comparison of the glow curves of TLD-100 (LiF: Mg, Ti) with the topaz/corundum composite sintered at 975 °C and 0 min.

Fig. 23 compares the TLD-100 glow curve' emission with that of the composite that presented the best thermoluminescent response sintered at 975 °C for 0 min (best treatment). The emission intensities of the topaz/corundum composite are above that of the commercial dosimeter. The peak position for both materials varies; the useful peak of the composite (peak 3) occurs at 221 °C while peak 5 of the TLD-100 is at 210 °C. The composite presents peaks at higher temperatures being more stable since they are less susceptible to fading at room temperature than those at lower temperatures.

In personal and clinical dosimetry, the dosimeter must have a behavior similar to human tissue and an effective atomic number close to 7.4 (Z_eff_ human tissue) [50]. The topaz/corundum composite with the best TL response (975 °C, 0 min) presents a Z_eff_ of 11.74, 1.6 times higher than that for human tissue. Based on this parameter, regarding dosimetric applications, the topaz/corundum composite sintered in this work does not score before the commercial TLD-100 dosimeter. However, the composite can be a good choice for medical, geological dating, and environmental dosimetry.

**Fig. 24:**
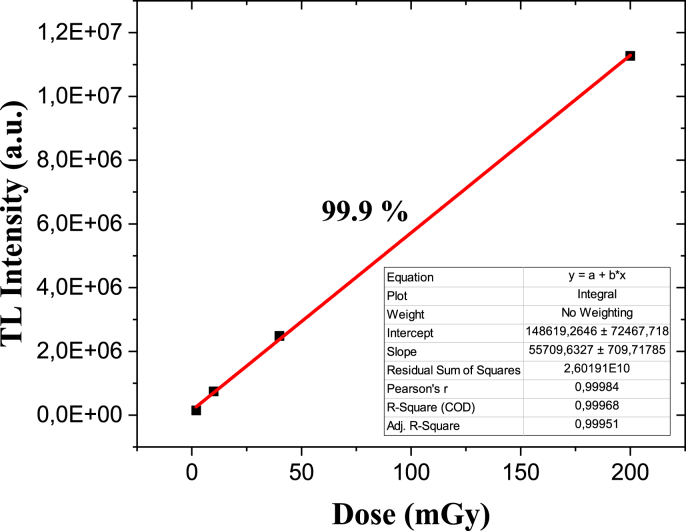
TL response as a function of dose (mGy) of topaz/corundum composite sintered at 975 °C for 0 min.

Personal dosimeters are used in the radiological inspection of external exposures such as the whole body, extremities, and specific areas received by a worker exposed to ionizing radiation, obtained by reading the dosimeter assigned and worn by the person throughout the working day. An analysis of radiation detection at lower doses was performed. Fig. 24 shows the thermoluminescent intensity of the sintered composite topaz/corundum that obtained the most useful peak (925 °C and 0 min) as a function of dose in mGy. The composite presented a linear response with an adjustment of 99.9 % from 2 mGy to 200 mGy. In this study, the detection of lower doses in topaz/corundum composites suggests their possible application in radiotherapy.

**Fig. 25:**
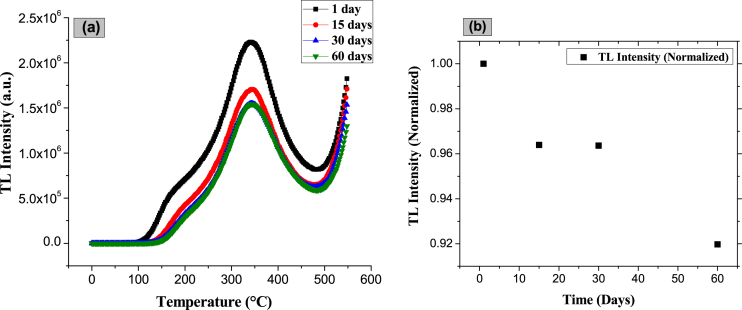
a) Glow curves of topaz/corundum composite measured at different times after irradiation and **b)** fading of glow curves as a function of time.

Fig. 25 presents the results of the glow curves (Fig. 25a) and the fading graph determined at different time intervals (1, 15, 30, and 60 days) of the topaz/corundum composite that presented the best TL response. The TL signal decrease was calculated by comparing the intensity of the samples measured after 1 irradiation day. After 15 days, there was a decrease of 4 % in the initial value of the glow curve's response total area, remaining constant for up to 30 days. After 60 days, the signal's decrease was 8 % (Fig. 25b), below the 10 % limit acceptable in a 30-day monitoring period for dosimetric applications, according to the IEC Standard (International Electrotechnical Commission) [51]. From the glow curves in Fig. 25a, it is seen that the shoulder located between 100 and 200 °C decreases as storage time elapses until it fades completely at 60 days. The greatest fading in the composite is due to surface traps corresponding to peaks at low temperatures.

Trap parameters such as kinetic order (*b*), activation energy (*E*), and frequency factor (*s*) of glow curves’ peaks were determined using the glow peak shape method proposed by Chen [52]. This method is one of the most widely used to determine the activation energy since it allows for obtaining reliable results from experimentally obtained glow curves [53]. Analytical equations relating the geometrical characteristics of the shape of TL peaks, corresponding to a second-order kinetics as proposed by Grossweiner [54], Lushchik [55], Halperin y Braner [56], Balarin [57], and Chen [58], were used. Table 2, Table 3 show the analysis results of the topaz/corundum composite analysis that presented the best treatment (975 °C and 0 min). The TL peaks of the composite present a second-order kinetics with similar values between of the activation energies of the capture centers and lower values of the frequency factor due to a higher amount of trapped electron release per unit time.

**Table 2:** Geometric parameters used for the determination of *b*, *E*, and *s* of the topaz/corundum composite sintered at 975 °C and 0 min.

Peak	T_1_ (°**C**)	T_2_ (°**C**)	T_M_ (°C)	τ	δ	ω	$\mu_{g}$	*b*	*s* (s^−1^)
1	84.06	141.24	112.11	28.05	29.13	57.18	0.51	**2**	**2.45x10**^**12**^
2	119.36	188.85	152.46	33.10	36.39	69.49	0.52	**2**	**5.61x10**^**10**^
3	179.82	258.89	219.90	40.08	38.99	79.07	0.49	**2**	**2.51x10**^**10**^
4	239.60	298.43	268.90	29.30	29.53	58.83	0.50	**2**	**8.67x10**^**7**^
5	244.66	337.41	291.86	47.20	45.55	92.75	0.49	**2**	**2.30x10**^**8**^

**Table 3:** Activation Energy (*E*, eV) of the topaz/corundum composite sintered with best treatment.

Method	Peak 1	Peak 2	Peak 3	Peak 4	Peak 5
Grossweiner [54]	0.71	0.73	0.81	1.37	0.90
Lushchik [55]	0.75	0.73	0.92	1.46	1.03
Halpering **&** Braner [56]	0.69	0.71	0.78	1.38	0.86
Balarin [57]	0.73	0.74	0.87	1.41	0.97
Chen [58]	0.68	0.71	0.74	1.34	0.82
Chen [58]	0.72	0.74	0.81	1.35	0.90
Chen [58]	0.70	0.73	0.78	1.35	0.87

The results show that the sintered topaz/*in situ* corundum composites with best thermoluminescent response at 975 °C and 0 min (best treatment) of the present study outperform the linear response range of topaz materials previously reported by the same research group at CINVESTAV-Saltillo, with a linear behavior from 0.02 to 66 Gy with 97.7 % adjustment [13,29]. The wide-range linear dose-response vs. TL intensity of the composites suggests the potential for low (mGy)- and high-dose (up to 300 Gy) dosimetry with potential applications in medical, geological dating, and environment. Nonetheless, further studies correlating the physical, mechanical, and thermoluminescent properties with the microstructure to increase the linear response and sensitivity of the dosimeters remain advisable.

## 4 Summary and conclusions

Sintering temperature and dwell time are crucial parameters in the composites’ phase stability. At low temperatures (825 and 875 °C) and short times (0–20 min), a biphasic material (T-F) consisting of topaz (Al_2_F_1·44_(OH)_0·56_SiO_4_) and aluminum fluoride (AlF_3_) remains. With time increase (40–80 min) at 875 °C a corundum formation reaction occurs, obtaining the topaz-Al_2_F_1·44_(OH)_0·56_SiO_4_/corundum-Al_2_O_3_ composites with an *in situ* corundum percentage of 39.2 wt %. Increasing sintering temperatures (925 and 975 °C) leads to a higher corundum formation in the topaz matrix, with a maximum amount of 53 wt % at 925 °C (for 40 min) and 78.4 wt % at 975 °C (for 60 min). However, at longer residence times, due to topaz reconversion, the corundum percentage decreases. Thus, a new topaz reconversion reaction is proposed in this work. The highest density of 3.13 g/cm^3^ (11 % porosity) was for the biphasic material topaz-aluminum fluoride (T-F) sintered at 825 °C for 0 min. For the topaz/corundum composites, while the maximum density was 3.05 g/cm^3^ (17 % porosity) for specimens sintered at 925 °C for 20 min, the lowest density was 2.16 g/cm^3^ (42 % porosity) for those sintered at 975 °C for 80 min.

The glow curves of the topaz/*in situ* corundum composite sintered at 975 °C and 0 min dwell time show thermoluminescent peaks located within the characteristic temperature range (180–250 °C) for dosimetric applications. Moreover, computational deconvolution showed that the most helpful peak in the topaz/corundum composite's glow curves is located at 221 °C; it is sharp, exhibits the highest thermoluminescent response, and shows a linear behavior for doses in the range of 5 Gy–300 Gy with an adjustment of 99.8 %. The thermoluminescent response improvement of the topaz/corundum composites is attributed to the corundum formed *in situ* during sintering. Compared to the TLD-100 (LiF: Mg, Ti) commercial dosimeter, the composite's most helpful peak (peak 3) occurs at a slightly higher temperature, making it more stable, although the TLD-100 dosimeter has better sensitivity than the composite. A second dose-response analysis revealed linearity from 2 mGy to 200 mGy, with an adjustment of 99.9 %.

Fading rate studies of the composite with the best sintering treatment revealed a signal decrease of 4 % after 15 days, remaining constant for up to 30 days. After 60 days, the signal decreased by 8 %, which is within the limit acceptable value of 10 % in a 30-day monitoring period for dosimetric applications. The kinetic parameters, kinetics order (*b*), activation energy (*E*), and frequency factor (*s*) of traps present in the topaz/corundum composite were determined using the glow peak shape method. Following second-order kinetics, similar values of the activation energies of the capture centers for all the peaks were observed. The topaz/corundum composite with the best TL response (975 °C, 0 min) presents an effective atomic number (Z_eff_) of 11.74, 1.6 times higher than that for human tissue. The detection of lower doses (mGy) and the linear response at higher doses (Gy) of beta ^90^Sr, together with the other thermoluminescent properties, suggest that the topaz/corundum composites sintered at 975° for 0 min dwell time may find application in radiotherapy, geological dating, and environmental dosimetry.

## Data availability statement

All data generated or analyzed during this study are included in this published article.

## CRediT authorship contribution statement

**S.A. Sinclair:** Investigation, Formal analysis, Conceptualization. **M.I. Pech-Canul:** Supervision, Formal analysis, Conceptualization. **M.C. Acosta-Enríquez:** Investigation, Data curation. **R. Meléndrez Amavizca:** Software, Resources. **Alejandro Sala Crist:** Formal analysis. **J. Marcazzó:** Formal analysis.

## Declaration of competing interest

The authors declare that they have no known competing financial interests or personal relationshipsthat could have appeared to influence the work reported in this paper.

## References

[1] Sardar M., Souza D.N., Groppo D.P., Caldas L.V.E., Tufail M. (2013). Suitability of topaz glass composites as dosimeters using optically stimulated luminescence technique. IEEE Trans. Nucl. Sci..

[2] Rocha F.C.D., Oliveira M.L., Cecatti S.G.P., Caldas L.V.D. (2003). Properties of sintered amethyst pellets as thermoluminescent dosimeters. Appl. Radiat. Isot..

[3] Kaur R., Bhatia V., Kumar D., Rao S.M.D., Singh S.P., Kumar A. (2019). Physical, structural, optical and thermoluminescence behavior of Dy_2_O_3_ doped sodium magnesium borosilicate glasses. Results Phys..

[4] Cogollo P.R., Quintero J.S., Guitierrez Flores O. (2013). Dosimetric and thermoluminescent characteristics of sintered samples based on Li_2_OAl_2_O_3_nSiO_2_ systems. Rev. Mexic. Fisica.

[5] Salama E., Soliman H.A., Youssef G.M., Hamad S. (2017). Thermoluminescence properties of borosilicate glass doped with ZnO. J. Lumin..

[6] Aboud H., Wagiran H., Hussin R., Ali H., Alajerami Y., Saeed M.A. (2014). Thermoluminescence properties of the Cu-doped lithium potassium borate glass. Appl. Radiat. Isot..

[7] Souza D.N., Meira R.A., Lima J.F., Valerio M.E.G., Caldas L.V. (2003). Evaluation of doses in radiotherapy using solid-state composites based on natural colourless topaz. Appl. Radiat. Isot..

[8] Moss A.L., McKlveen J.W. (1978). Thermoluminescent properties of topaz. Health Phys..

[9] Yukihara E.G., Okuno E. (1998). On the thermoluminescent properties and behaviour of Brazilian topaz. Nucl. Instrum. Methods Phys. Res., Sect. B: Beam Interactions wirh Materials and Atoms.

[10] Sardar M., Tafail M. (2011). Thermoluminescent characteristics of topaz from Sabser mine near Sakardu in northern Pakistan. Nucl. Instrum. Methods Phys. Res., Sect. B: Beam Interactions wirh Materials and Atoms.

[11] Nieto J.A. (1979).

[12] Trujillo-Vázquez E., Pech-Canul M.I. (2015). Formation pathway, structural characterization and optimum processing parameters of synthetic topaz–Al_2_SiO_4_ (OH, F)_2_–by CVD. J. Solid State Chem..

[13] Trujillo-Vázquez E., Pech-Canul M.I., Marcazzó J. (2016). Thermoluminescent characterization of Al_2_O_3_-derived synthetic topaz. J. Alloys Compd..

[14] Trujillo-Vázquez E., Pech-Canul M.I., Marcazzó J. (2017). Topaz synthesis using Al_2_O_3_, Al(OH)_3_ or Al_2_Si_2_O_5_(OH)_4_ and color centers promoting its radioluminescence response. J. Alloys Compd..

[15] Camargo L., Trujillo-Vázquez E., Pech-Canul M.I., Marcazzó J. (2017). OSL dosimetric properties of synthetic topaz. Radiat. Meas..

[16] Trujillo-Vázquez E., Pech-Canul M.I. (2015). Topaz and some materials with thermoluminescent properties and potential applications in dosimetry. MRS Online Proc. Libr..

[17] Schober R., Thilo E. (1940). Chemische Untersuchungen von Silicaten, X. Mitteil. Über den Topas Al_2_SiO_4_(F,OH,O)_2_ und seine Synthese und über ein neues fluorhaltiges Aluminiumoxyd. Ber. Dtsch. Chem. Ges..

[18] Coes L. (1955). High‐pressure minerals. J. Am. Ceram. Soc..

[19] Rosenberg P.E. (1972). Compositional variations in synthetic topaz. Am. Mineral.: Journal of Earth and Planetary Materials.

[20] Adbel Rehim A.M. (1975). Formation of mullite, topaz and corundum. Thermochim. Acta.

[21] Sōmiya S., Hirano S., Yoshimura M., Shima H. (1989). Hydrothermal synthesis of topaz crystals. Hydrothermal Reactions for Materials Science and Engineering.

[22] Teixeira M.I., Caldas L.V.E. (2004). Sintered sand pellets for high-dose dosimetry. Nucl. Instrum. Methods Phys. Res. Sect. B Beam Interact. Mater. Atoms.

[23] Caldas L.V., Teixeira M.I., Ferraz G.M. (2006). Influence of thermal treatments on the response of sand radiation detectors for high-dose dosimetry. Radiation proteccion dosimetry.

[24] Souza D.N., Melo A.P., Oliveira M.G., Caldas L.V. (2007). Dosimetric characterization of wollastonite‐teflon composites. Phys. Status Solidi C.

[25] Teixeira M.I., Caldas L.V.E. (2014).

[26] Souza D.N., Lima J.F., Valerio M.G., Caldas L.V.E. (2002). Performance of pellets and composites of natural colourless topaz as radiation dosemeters. Radiat. Protect. Dosim..

[27] De Magalhaes C.M.S., Souza D.N., Caldas L.V. (2006). Use of composites of topaz–glass as TSEE and TL dosemeters. Radiat. Protect. Dosim..

[28] Bomfim K.S., Souza D.N. (2010). Applicability of topaz composites to electron dosimetry. J. Phys. Conf..

[29] Leal-Cruz A.L., Pech-Canul M.I. (2006). *In situ* synthesis of Si_3_N_4_ from Na_2_SiF_6_ as a silicon solid precursor. Mater. Chem. Phys..

[30] Leal-Cruz A.L., Pech-Canul M.I., Lara-Curzio E., Trejo R.M., Peascoe R. (2009). Selective synthesis and characterization of HYSYCVD-Si_2_N_2_O. Mater. Chem. Phys..

[31] Trujillo-Vázquez E., Pech-Canul M.I., Guía-Tello J.C., Pech-Canul M.A. (2016). Surface chemistry modification for elimination of hydrophilic Al_4_C_3_ in B_4_C/Al composites. Mater. Des..

[32] Sinclair S.A., Pech-Canul M.I., Trujillo-Vázquez E., Acosta-Enríquez M.C., Saavedra G., Acosta Enríquez E.B., González L.A., López-Cuevas J. (2020). Microstructure, properties and thermoluminescent response of sintered synthetic topaz (Al_2_SiO_4_F_1. 44_ (OH)_0.56_)/Al_2_O_3_ compounds. Mater. Today Commun..

[33] Sinclair S.A., Pech-Canul M.I. (2022). Development feasibility of TLD phosphors and thermoluminescent composite materials for potential applications in dosimetry: a review. Chem. Eng. J..

[34] Julien C.M., Mauger A. (2019). Functional behavior of AlF_3_ coatings for high-performance cathode materials for lithium-ion batteries. AIMS Materials Science.

[35] Riello D., Zetterström C., Parr C., Braulio M.A.L., Moreira M., Gallo J.B., Pandolfelli V.C. (2016). AlF_3_ reaction mechanism and its influence on α-Al_2_O_3_ mineralization. Ceram. Int..

[36] Leal-Cruz A.L., Pérez-Aguirre A., Meléndez-Amavizca R., Vera-Marquina A., Barboza-Flores M., Pech-Canul M.I., Hernandez-Torres J., Dominguez-Chavez J.G., Martínez-Castillo J. (2016). HYSYCVD synthesis of 1D nanostructures of TiO_2_. Nanosci. Nanotechnol..

[37] Leal-Cruz A.L., Pech-Canul M.I. (2007). Synthesis of Si_3_N_4_ from Na_2_SiF_6_as a solid precursor: microstructural evolution. Mater. Sci. Forum.

[38] Leal-Cruz A.L., Pech-Canul M.I., De La Peña J.L. (2008). A low-temperature and seedless method for producing hydrogen-free Si_3_N_4_. Rev. Mexic. Fisica.

[39] Flores-García J.C., Pech-Canul M.I., Leal-Cruz A.L., Rendón-Angeles J.C. (2012). Synthesis of (α-and β-) Si_3_N_4_/Si_2_N_2_O into silicon particulate porous preforms by hybrid system CVI and direct nitridation. J. Eur. Ceram. Soc..

[40] Acosta-Enriquez E.B., Acosta-Enriquez M.C., Castillo-Ortega R., Zayas M.A.E., Pech-Canul M.I. (2016). Nanostructured fibers of A-SI3N4 deposited by HYSY-CVD. Dig. J. Nanomater. Biostruct..

[41] Soltani N., Bahrami A., Pech-Canul M.I., González L.A., Gurlo A. (2018). Surface modification of rice-husk ash (RHA) by Si_3_N_4_ coating to promote its wetting by Al-Mg-Si alloys. Mater. Chem. Phys..

[42] Leal-Cruz A.L. (2004).

[43] Chung F.H. (1974). Quantitative interpretation of X-ray diffraction patterns of mixtures. I. Matrix-flushing method for quantitative multicomponent analysis. J. Appl. Crystallogr..

[44] Hughes S.W. (2006). Measuring liquid density using Archimedes' principle. Phys. Educ..

[45] Abdel-Rehim A.M. (1999). Thermal analysis of topaz synthesis from kaolinite. Thermochim. Acta.

[46] Souza D.N., De Lima J.F., Valerio M.E.G., Caldas L.V.E. (2006). Thermally stimulated luminescence and EPR studies on topaz. Appl. Radiat. Isot..

[47] McKeever S.W.S. (1991). Mechanisms of thermoluminescence production: some problems and a few answers?. Int. J. Radiat. Appl. Instrum. Nucl. Tracks Radiat. Meas..

[48] Biderman S., Horowitz Y.S., Oster L., Einav Y., Dubi Y. (2002). Glow curve analysis of composite peak 5 in LiF: Mg, Ti (TLD-100) using optical bleaching, thermal annealing and computerised glow curve deconvolution. Radiat. Protect. Dosim..

[49] Nieto J.A. (1993).

[50] Ranogajec-Komor M. (2005).

[51] International Electrotechnical Commision. Internaltional Standard, CEI IEC 1066, 1991-12. Thermoluminescence Dosimetry Systems for Personal and Environmental Monitoring..

[52] Chen R. (1969). On the calculation of activation energies and frequency factors from glow curves. J. Appl. Phys..

[53] Borbon Nuñez H.A. (2010).

[54] Grossweiner L.I. (1953). A note on the analysis of first‐order glow curves. J. Appl. Phys..

[55] Lushchik C.B. (1956). The investigation of trapping centers in crystals by the method of thermal bleaching. Soviet Physics Jetp-USSR.

[56] Halperin A., Braner A.A. (1960). Evaluation of thermal activation energies from glow curves. Phys. Rev..

[57] Balarin M. (1979). Half-width and asymmetry of glow peaks and their consistent analytical representation. J. Therm. Anal..

[58] Chen R. (1976). Methods for kinetic analysis of thermally stimulated processes. J. Mater. Sci..

